# Ultrasonographic and computed tomographic findings of an intra-abdominal lipoma in a dog

**DOI:** 10.1007/s11259-026-11071-0

**Published:** 2026-01-20

**Authors:** Maria Paula Luchi da Silva Mattos, Luiz Caian Stolf, Rafael Kretzer Carneiro

**Affiliations:** Department of Veterinary Medicine, State University of Santa Catarina (UDESC), Av. Luiz de Camões, Bairro Conta Dinheiro, SC Lages, Brazil

**Keywords:** Canine, Neoplasia, Imaging exams, Cytology

## Abstract

The aim of this study was to describe the ultrasonographic and computed tomographic findings of a rare case of an atypical lipoma located in the abdominal cavity of a 13-year-old neutered male Schnauzer weighing 10.3 kg. The animal had a history of dyslipidemia and hypothyroidism, and, due to this clinical background, additional diagnostic tests were requested. On B-mode ultrasonography, an amorphous, hyperechoic, and homogeneous structure with well-defined borders was visualized in the mid-abdominal region, measuring approximately 10.3 cm in length and 6.8 cm in height. Subsequently, interventional ultrasonography was performed to obtain a guided sample. Because of the difficulty in delineating and evaluating adjacent organs, the animal was referred for computed tomography, which identified a well-defined expansile mass with smooth margins and homogeneous density, exhibiting attenuation values consistent with adipose tissue. The integration of imaging modalities, combined with image-guided sampling, proved essential for complementing the clinical assessment and guiding the diagnostic process.

## Background

Adipose masses, particularly lipomas, are relatively frequent conditions in veterinary medicine (Kurihara et al. 2018). Lipomas are benign neoplasms of mesenchymal origin, composed of differentiated adipocytes, which in most cases are clinically insignificant (O’Neill et al. 2018). A lipoma is characterized as a soft, encapsulated, and well-defined mass, with a low recurrence rate following surgical excision (Kurihara et al. 2018). Lipomas are more frequently diagnosed in dogs (Dhein et al. 2024), which present an approximately 3.3-fold higher risk of developing the condition compared with cats (Juodžiukynienė 2025). In dogs, these neoplasms predominantly occur in the abdomen, flanks, thoracic and axillary regions, neck, and scapular region, whereas in cats the most common locations include the flanks, back, and abdomen (Juodžiukynienė 2025).

Although the majority of lipomas are located in subcutaneous tissue, there are also reports of intrathoracic, intra-abdominal, and intrapelvic occurrences, which are considered atypical locations and rare (Daleck and Nardi 2016; Matsuyama et al. 2025). When located within body cavities, lipomas may remain asymptomatic for long periods and can reach considerable dimensions before causing clinical manifestations. In such cases, the observed signs are usually associated with the compression or entrapment of adjacent organs (Kim et al. 2017). Although they may remain undetectable for extended intervals, when identified, they are often of substantial size. Surgical intervention is recommended only when there is impairment of normal organic functions (Daleck and Nardi 2016).

Extracavitary lipomas can be diagnosed with fine needle aspiration and cytology, including ultrasonography and ultrasound-guided sampling (Lamagna et al. 2012). Intracavitary lipomas can be diagnosed with transabdominal ultrasonography (US) or abdominal Computed Tomography (CT) and Magnetic Resonance Imaging (MRI) for advanced cases (Komi et al. 2024). However, differentiating lipomas from obesity-related fat deposits can be challenging in CT examinations. In most cases, lipomas appear as homogeneous fat-density lesions, showing little to no enhancement on post-contrast images (Kim et al. 2017). Abdominal radiography can also be used, in which it appears as a mass with radiopacity typical of fat. On ultrasonography, it is observed as a hyperechoic and poorly vascularized mass (Clapp et al. 2009).

In this context, the aim of the present study is to report the case of a 13-year-old male dog diagnosed with an intra-abdominal lipoma through a combination of imaging examinations, including computed tomography and ultrasonography, complemented by cytological evaluation.

## Case presetation

A 13-year-old neutered male Schnauzer weighing 10.3 kg was evaluated with a medical history of dyslipidemia and hypothyroidism. The patient was maintained on a low-fat diet and was receiving oral pharmacologic therapy with bezafibrate (10 mg/kg, SID) and levothyroxine sodium (10 µg/kg, BID). Based on the clinical history, hematologic and biochemical tests were performed, which revealed increased alanine aminotransferase (ALT, 104 U/L) and alkaline phosphatase (1567 U/L) activities, as well as elevated serum cholesterol (456 mg/dL) and triglyceride concentrations (228 mg/dL). No additional laboratory abnormalities were detected.

**Fig. 1:**
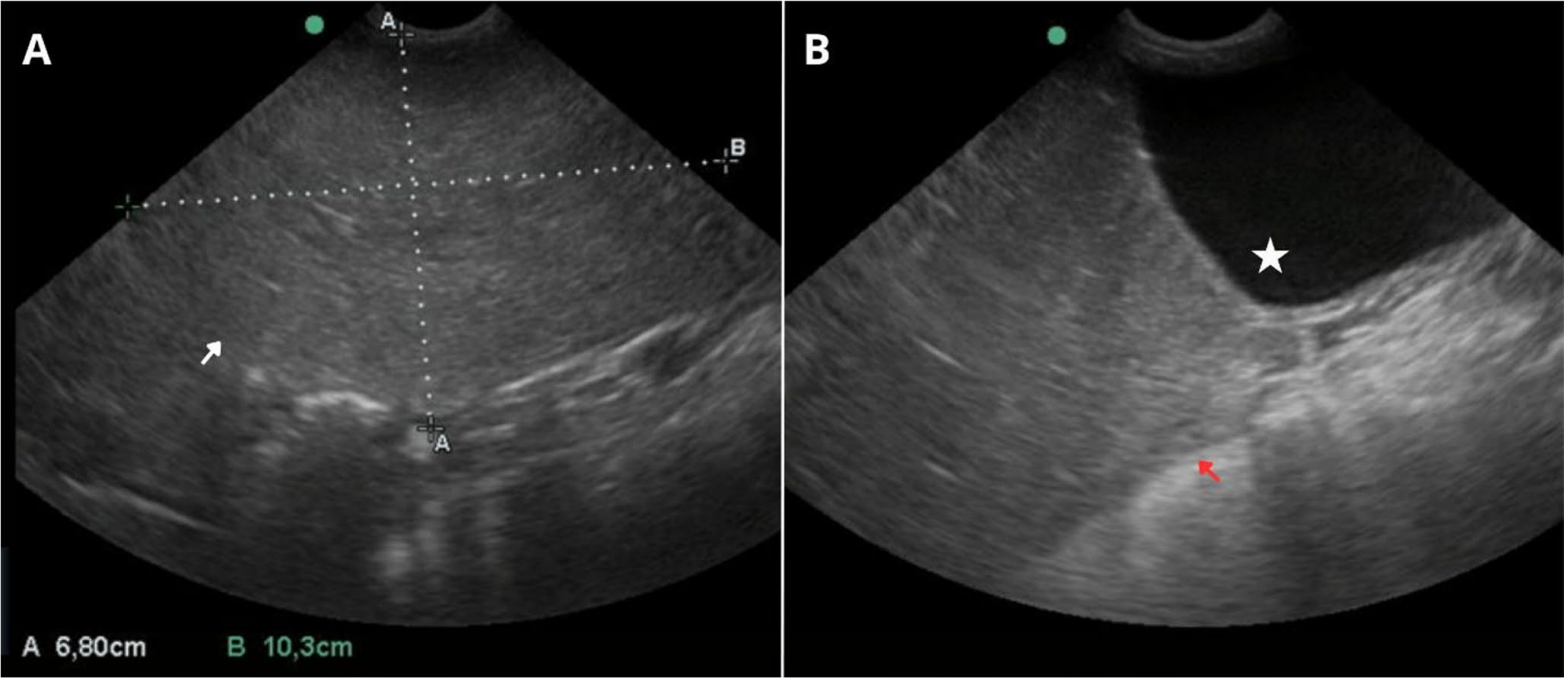
Abdominal ultrasonography of a dog presenting with an abdominal mass. A well-defined, homogeneous, hyperechoic structure (white arrow) measuring approximately 6.8 cm (height) × 10.3 cm (length) is observed (**A**). The identified mass (red arrow) is in close contact with the urinary bladder (white star), but its complete volumetric delineation cannot be assessed (**B**)

The ultrasonographic examination was performed using a Sonosite Edge II system (Fujifilm, Bothell, WA, USA) equipped with multifrequency convex and linear transducers. An amorphous, hyperechoic, homogeneous structure with well-defined margins was identified in the mid-abdominal region, measuring approximately 10.3 cm in length and 6.8 cm in height (Fig. 1). No evidence of peritoneal reactivity, free fluid, or compression of adjacent organs was observed. Based on the ultrasonographic findings, an ultrasound-guided fine-needle aspiration was performed, which revealed moderate cellularity characterized by large clusters of adipocytes with abundant clear cytoplasm and peripherally displaced pyknotic nuclei findings consistent with a lipoma.

**Fig. 2:**
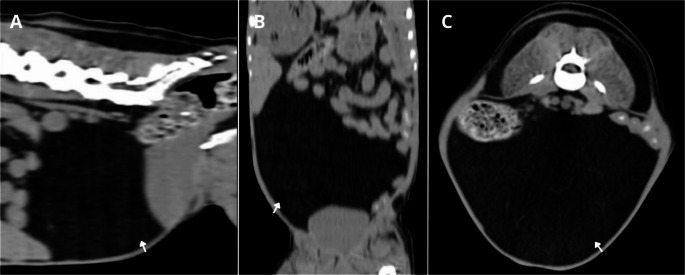
Computed tomography of the abdomen of a dog. In sagittal (**A**), coronal (**B**), and axial (**C**) images, a hypoattenuating structure (white arrows) with well-defined margins and homogeneous appearance can be identified occupying the caudal abdominal region, consistent with adipose tissue (reaching up to − 117 Hounsfield Units)

To better delineate the size of the structure and assess potential secondary involvement, a computed tomography (CT) scan was performed (Toshiba Activion, 16-slice) using a slice thickness of 1.3 mm. The examination revealed a well-defined expansile mass with smooth margins and homogeneous density, exhibiting attenuation values consistent with adipose tissue (reaching up to − 117 Hounsfield Units). The lesion was located in the ventromediocaudal region of the abdomen (Fig. 2). No contrast enhancement was observed following intravenous administration of iodinated contrast medium, and there were no signs of invasion or compression of adjacent organs. Following CT evaluation, surgical removal of the mass was recommended; however, the procedure was not authorized by the owners.

### Discussion and conclusions

The clinical findings observed in this case, combined with diagnostic imaging examinations and cytologic evaluation, were fundamental in guiding the investigation and supporting the diagnostic suspicion of an atypical intra-abdominal lipoma. Although lipomas are commonly identified within subcutaneous tissue (Morgan 2025), the occurrence of atypical variants, particularly those located intra-abdominally, is considered uncommon (Lappalainen et al. 2025).

Although the authors are not aware of any direct correlation between dyslipidemia and the development of lipomas in dogs, whether subcutaneous or intra-abdominal, both conditions may share obesity as a predisposing factor (O’Neill et al. 2018). Obesity is known to significantly alter lipid metabolism and is simultaneously associated with an increased occurrence of lipomas (Jeusette et al. 2025). Thus, although a causal relationship between dyslipidemia and lipomas remains undefined, it is plausible to consider a potential indirect pathophysiologic link mediated by excessive body adiposity.

Diagnostic imaging techniques are essential for the detection, characterization, and monitoring of abdominal masses, with ultrasonography, given its noninvasive, painless, and widely accessible nature, serving as the primary screening tool for the initial identification of neoplasms (Ercolin et al. 2024). In the present report, B-mode ultrasonography supported clinical decision making; however, due to the large extent of the mass, precise delineation was not achievable, which also hindered adequate assessment of the adjacent structures.

CT allowed correlation of the ultrasonographic findings and provided a more accurate volumetric assessment of the lesion, an aspect that cannot be achieved with ultrasonography. Unlike ultrasound, CT offers high spatial resolution and superior tissue contrast, enabling improved delineation of the mass, its extent, and its anatomical relationship with adjacent structures. This capability is particularly relevant in intra-abdominal neoplasms, in which determining margins and potential tissue invasion is critical, yet could not be established on ultrasonographic examination (O’Mara et al. 2025).

Although the imaging findings contributed substantially to the morphological characterization of the lesions, they were insufficient to determine the type of cellularity involved, thereby requiring complementary cytologic and histopathologic evaluation. In cases of intra-abdominal masses, ultrasound-guided sampling represents a safe and minimally invasive approach, enabling targeted collection of material (Holter et al. 2023). In the present report, the use of interventional ultrasonography for sample collection allowed the establishment of a diagnosis of lipoma, demonstrating its utility as a minimally invasive method for abdominal assessment. Furthermore, the image-guided technique not only facilitated precise and safe sampling but also reduced the risk of complications when compared with open surgical techniques for obtaining diagnostic material.

This report presents several limitations inherent to the evaluation of a single animal, which restricts the generalizability of the findings. Additionally, the underlying cause of the mass could not be determined, as no complementary tests were performed that would allow full elucidation of its etiology. The absence of surgical intervention also limited the precise assessment of prognosis, thereby constraining a more comprehensive understanding of the patient’s clinical course.

## Data Availability

No datasets were generated or analysed during the current study.
